# (*E*)-*N*-[2-(Benzyl­iminometh­yl)phen­yl]-2,6-diisopropyl­aniline

**DOI:** 10.1107/S1600536809042202

**Published:** 2009-10-17

**Authors:** Yi-Chang Liu, Chia-Her Lin, Hsiao-Li Chen, Bao-Tsan Ko

**Affiliations:** a Department of Chemistry, Chung Yuan Christian University, Chung-Li 320, Taiwan; b Department of Chemistry, National Chung Hsing University, Taichung 402, Taiwan

## Abstract

The mol­ecular conformation of the title compound, C_26_H_30_N_2_, is reinforced by an intra­molecular N—H⋯N hydrogen bond, resulting in an almost planar [mean deviation of 0.023 (2) Å] *S*(6) ring. The dihedral angles between the central benzene ring and the terminal unsubstituted and substituted aromatic rings are 64.45 (9) and 89.40 (8)°, respectively.

## Related literature

For background information on anilido-aldimine ligands, see: Lee *et al.* (2005 ▶); Yao *et al.* (2008 ▶). For related structures: see: Gao *et al.* (2008 ▶); Tsai *et al.* (2009 ▶).
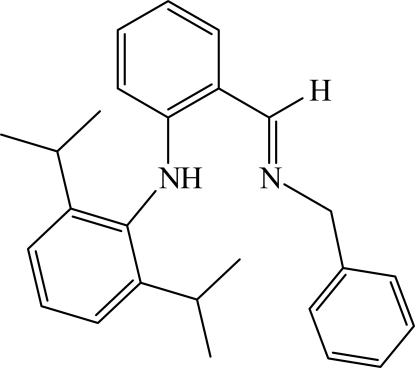


## Experimental

### 

#### Crystal data

C_26_H_30_N_2_
*M* *_r_* = 370.52
Triclinic, 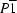

*a* = 8.0583 (3) Å
*b* = 10.9186 (4) Å
*c* = 13.5974 (5) Å
α = 75.823 (2)°
β = 77.933 (2)°
γ = 82.709 (2)°
*V* = 1130.68 (7) Å^3^
*Z* = 2
Mo *K*α radiation
μ = 0.06 mm^−1^
*T* = 296 K
0.53 × 0.46 × 0.32 mm

#### Data collection

Bruker APEXII CCD diffractometer
Absorption correction: multi-scan (*SADABS*; Bruker, 2008 ▶) *T*_min_ = 0.965, *T*_max_ = 0.979
24959 measured reflections
5557 independent reflections
3150 reflections with *I* > 2σ(*I*)
*R*_int_ = 0.061

#### Refinement

*R*[*F* ^2^ > 2σ(*F* ^2^)] = 0.054
*wR*(*F* ^2^) = 0.178
*S* = 1.00
5557 reflections
253 parameters
H-atom parameters constrained
Δρ_max_ = 0.22 e Å^−3^
Δρ_min_ = −0.18 e Å^−3^

### 

Data collection: *APEX2* (Bruker, 2008 ▶); cell refinement: *SAINT-Plus* (Bruker, 2008 ▶); data reduction: *SAINT-Plus*; program(s) used to solve structure: *SHELXS97* (Sheldrick, 2008 ▶); program(s) used to refine structure: *SHELXL97* (Sheldrick, 2008 ▶); molecular graphics: *SHELXTL* (Sheldrick, 2008 ▶); software used to prepare material for publication: *SHELXTL*.

## Supplementary Material

Crystal structure: contains datablocks I, global. DOI: 10.1107/S1600536809042202/hb5139sup1.cif

Structure factors: contains datablocks I. DOI: 10.1107/S1600536809042202/hb5139Isup2.hkl

Additional supplementary materials:  crystallographic information; 3D view; checkCIF report

## Figures and Tables

**Table 1:** Hydrogen-bond geometry (Å, °)

*D*—H⋯*A*	*D*—H	H⋯*A*	*D*⋯*A*	*D*—H⋯*A*
N2—H2*A*⋯N1	0.86	2.03	2.7113 (15)	136

**Fig. 1.**
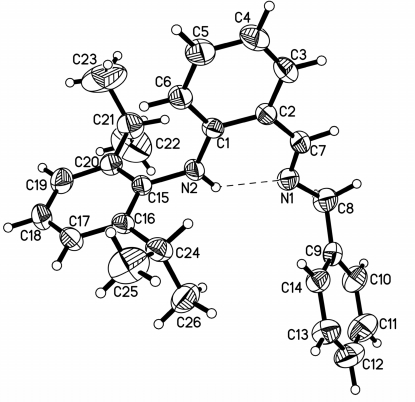
A view of the molecular structure of (I) with displacement ellipsoids drawn at the 30% probability level. H atoms are presented as the small spheres of arbitrary radius.

**Fig. 2.**
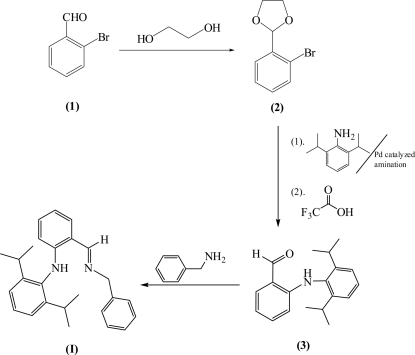
Reaction scheme leading to the title compound.

## supplementary crystallographic information

### Comment

Recently, anilido-aldimine (AA) metal compounds have been attracting considerable attention, mainly due to their applications in the catalytic polymerization (Lee *et al.*, 2005 & Yao *et al.*, 2008). These AA ligands can be designed to control the steric or electronic effect to provide a single active metal center for minimizing the side reaction. For instance, a series of NNN-tridentate AA rare-earth metal complexes has been demonstrated that the pendant arm of the quinolinyl group can coordinate with the metal to increase the sterics and coordination sites of the ligand, creating a single active site nature to initiate the polymerization of ε-caprolactone (ε-CL). (Gao *et al.*, 2008). Most recently, *Tsai *et al.**, (2009) have successfully synthesized and structural characterized the Mg (II) and Zn (II) complexes supported from a novel NNN-tridentate AA with the pendant arm on the imine nitrogen and have demonstrated their catalytic studies of ring-opening polymerization of ε-CL and *L*-lactide. Therefore, our group is interested in developing new approaches for the synthesis of bi- or multi-dentate AA from the substituted benzaldehyde derivatives. Herein, we report the synthesis and crystal structure of the title compound, (I), a potential NN-bidentate AA ligand for the preparation of aluminium, magnesium and zinc complexes (Scheme 1).

The solid structure of (I) reveals the phenyl configuration containing one 2,6-diisopropylphenylamino functionalized group and one benzyl substituted imine group on the *ortho* position (Fig. 1). It was found that there is an intramolecular N—H···N hydrogen bond between the amine and imine groups (Table 1). It is interesting to note that the six-member ring (N1, C7, C2, C1, N2, H2A) formed from the N—H···N hydrogen-bond is almost planar with a mean deviation of 0.023 (2) Å.

### Experimental

The title compound (I) was synthesized by the following procedures (Fig. 2):

2-(2-Bromophenyl)-1, 3-dioxolane (2). In a 50 ml two-necked round-bottomed flask, a solution of 2-bromobenzaldehyde, 1 (20.0 g, 108.0 mmol) in 40 ml of toluene was added in one portion anhydrous ethylene glycol (8.69 g, 140.0 mmol) and *p*-toluenesulfonic acid (186 mg, 1.08 mmol). The resulting solution mixture was refluxed until the theoretical yield of water had been collected in a Dean–Stark trap. After 16 h, the mixture was cooled, washed with 10% aqueous sodium hydroxide (2 *x* 30 ml), followed by deionized water (2 *x* 30 ml), brine (1 *x* 30 ml) and the organic layer was dried over anhydrous MgSO_4_. The solvent was removed *in vacuo* to give 24.18 g (94%) of a clear yellow viscous oil, 2. ^1^H NMR (CDCl_3_, p.p.m.): δ 7.18 - 7.60 (m, 4H, Ph*H*), 6.09 (s, 1H, PhC*H*), 4.03–4.17 (m, 4H, OC*H*_2_C*H*_2_O).

2-(2, 6-Diisopropyl-phenylamino)benzaldehyde (3). In a 250 ml two-necked round-bottom flask equipped with a magnetic stir bar and a condenser, Pd(*OAc*)_2_ (71 mg, 0.32 mmol), sodium *tert*-butoxide (6.15 g, 64.0 mmol) and 2-(2-bromophenyl)-1, 3-dioxolane, 2 (7.30 g, 32.0 mmol) was degassed by vacuum. Under N_2_ atmosphere, tri-*tert*-butylphosphane (129 mg, 0.64 mmol), 2, 6-diisopropylaniline (6.29 g, 35.0 mmol) and anhydrous tetrahydrofuran (50 ml) were added and refluxed for 16 h. The reaction mixture were then cooled to ambient temperature, filtered the resulting solution. The solution portion was extracted with ethyl acetate and deionized water washing twice, and the organic layer was dried over magnesium sulfate and the solvent was evaporated under vacuum. The crude product was dissolved in hexane (100 ml) and was cooled in 253 K overnight to obtain the brown solids (8.11 g, 78%)..^1^H NMR (CDCl_3_, p.p.m.): δ 7.39 (d, *J* = 7.5 Hz, 1H, Ph*H*), 7.30–7.20 (m, 3H, Ph*H*), 7.05 (t, *J* = 7.5 Hz, 1H, Ph*H*), 6.71 (t, *J* = 7.5 Hz, 1H, Ph*H*), 6.33 (s, 1H, PhN*H*), 6.18 (d, *J* = 8.1 Hz, 1H, Ph*H*), 5.99 (s, 1H, PhC*H*), 4.07–4.18 (m, 4H, OC*H*_2_C*H*_2_O), 3.14 (m, *J* = 6.9 Hz, 2H, C*H*(CH_3_)_2_), 1.16 (d, *J* = 6.9 Hz, 6H, CH(C*H*_3_)_2_), 1.12 (d, *J* = 6.9 Hz, 6H, CH(C*H*_3_)_2_). The above brown solids, 2-[2-(2, 6-diisopropylanilino)phenyl]-1,3-dioxolane (5.34 g, 16.4 mmol), trifluoroacetic acid (1.14 g, 10.0 mmol) and MeOH (30 ml) were added in the 100 ml round-bottom and the mixture was stirred at ambient temperature. After 1 h, the resulting solution was removed the solvent *in vacuo*. The residue was then extracted with ethyl acetate and deionized water washing twice. The final organic layer was dried over anhydrous MgSO_4_ and the solvent was removed under vacuum to give white solids (4.33 g, 94%). ^1^H NMR (CDCl_3_, p.p.m.): δ 9.97 (s, 1H, PhN*H*), 9.57 (s, 1H, PhC(O)*H*), 7.57 (d, *J* = 7.8 Hz, 1H, Ph*H*), 7.23–7.38 (m, 4H, Ph*H*), 6.74 (t, *J* = 7.5 Hz, 1H, Ph*H*), 6.25 (d, *J* = 8.4 Hz, 1H, Ph*H*), 3.08 (m, *J *= 6.9 Hz, 2H, C*H*(CH_3_)_2_), 1.18 (d, *J* = 6.9 Hz, 6H, CH(C*H*_3_)_2_), 1.12 (d, *J* = 6.9 Hz, 6H, CH(*CH*_3_)_2_).

(*E*)—*N*-(2-((benzylimino)methyl)phenyl)-2,6-diisopropylaniline (I). A mixture of benzylamine (0.33 ml, 3.0 mmol), 2-(2, 6-diisopropyl-phenylamino)benzaldehyde, 3 (0.76 g, 2.7 mmol) and anhydrous MgSO_4_(2.0 g) were stirred in reflux hexane (20 ml) for 12 h. Volatile materials were removed under vacuum to give the white solids. Yield: 0.75 g (75%). Colourless crystals were obtained from the saturated Et_2_O solution. ^1^H NMR (CDCl_3_, p.p.m.): δ 10.60 (s, 1H, PhN*H*), 8.61 (s, 1H, *H*C=N), 6.24–7.37 (m, 12H, Ph*H*), 4.87(s, 2H, C*H*_2_Ph), 3.14 (m,*J* = 6.9 Hz, 2H, C*H*(CH_3_)_2_), 1.20 (d, *J* = 6.9 Hz, 6H, CH(C*H*_3_)_2_), 1.10 (d,*J* = 6.9 Hz, 6H, CH(C*H*_3_)_2_).

### Refinement

The H atoms were placed in idealized positions and constrained to ride on their parent atoms, with C—H = 0.93 Å with *U*_iso_(H) = 1.2 *U*_eq_(C) for phenyl hydrogen; 0.96 Å with *U*_iso_(H) = 1.5 *U*_eq_(C) for CH_3_ group; 0.97 Å with *U*_iso_(H) = 1.2 *U*_eq_(C) for CH_2_ group; 0.98 Å with *U*_iso_(H) = 1.2 *U*_eq_(C) for CH group; N—H = 0.86 Å with *U*_iso_(H) = 1.2 *U*_eq_(C).

### Crystal data

**Table:**

C_26_H_30_N_2_	*Z* = 2
*M**_r_* = 370.52	*F*(000) = 400
Triclinic, *P*1	*D*_x_ = 1.088 Mg m^−^^3^
Hall symbol: -P 1	Mo *K*α radiation, λ = 0.71073 Å
*a* = 8.0583 (3) Å	Cell parameters from 9972 reflections
*b* = 10.9186 (4) Å	θ = 2.2–28.2°
*c* = 13.5974 (5) Å	µ = 0.06 mm^−^^1^
α = 75.823 (2)°	*T* = 296 K
β = 77.933 (2)°	Block, colourless
γ = 82.709 (2)°	0.53 × 0.46 × 0.32 mm
*V* = 1130.68 (7) Å^3^	

### Data collection

**Table:**

Bruker APEXII CCD diffractometer	5557 independent reflections
Radiation source: fine-focus sealed tube	3150 reflections with *I* > 2σ(*I*)
graphite	*R*_int_ = 0.061
Detector resolution: 8.3333 pixels mm^-1^	θ_max_ = 28.4°, θ_min_ = 1.6°
phi and ω scans	*h* = −10→10
Absorption correction: multi-scan (*SADABS*; Bruker, 2008)	*k* = −14→14
*T*_min_ = 0.965, *T*_max_ = 0.979	*l* = −18→18
24959 measured reflections	

### Refinement

**Table:**

Refinement on *F*^2^	Primary atom site location: structure-invariant direct methods
Least-squares matrix: full	Secondary atom site location: difference Fourier map
*R*[*F*^2^ > 2σ(*F*^2^)] = 0.054	Hydrogen site location: inferred from neighbouring sites
*wR*(*F*^2^) = 0.178	H-atom parameters constrained
*S* = 1.00	*w* = 1/[σ^2^(*F*_o_^2^) + (0.102*P*)^2^] where *P* = (*F*_o_^2^ + 2*F*_c_^2^)/3
5557 reflections	(Δ/σ)_max_ < 0.001
253 parameters	Δρ_max_ = 0.22 e Å^−^^3^
0 restraints	Δρ_min_ = −0.18 e Å^−^^3^

### Special details

**Table:**

Geometry. All e.s.d.'s (except the e.s.d. in the dihedral angle between two l.s. planes) are estimated using the full covariance matrix. The cell e.s.d.'s are taken into account individually in the estimation of e.s.d.'s in distances, angles and torsion angles; correlations between e.s.d.'s in cell parameters are only used when they are defined by crystal symmetry. An approximate (isotropic) treatment of cell e.s.d.'s is used for estimating e.s.d.'s involving l.s. planes.
Refinement. Refinement of *F*^2^ against ALL reflections. The weighted *R*-factor *wR* and goodness of fit *S* are based on *F*^2^, conventional *R*-factors *R* are based on *F*, with *F* set to zero for negative *F*^2^. The threshold expression of *F*^2^ > σ(*F*^2^) is used only for calculating *R*-factors(gt) *etc*. and is not relevant to the choice of reflections for refinement. *R*-factors based on *F*^2^ are statistically about twice as large as those based on *F*, and *R*- factors based on ALL data will be even larger.

### Fractional atomic coordinates and isotropic or equivalent isotropic displacement parameters (Å^2^)

**Table:**

	*x*	*y*	*z*	*U*_iso_*/*U*_eq_	
N1	0.46771 (17)	0.32146 (10)	0.01875 (8)	0.0622 (3)	
N2	0.45207 (13)	0.23379 (11)	0.22480 (8)	0.0585 (3)	
H2A	0.4023	0.2482	0.1725	0.070*	
C1	0.62287 (15)	0.24628 (12)	0.20617 (10)	0.0514 (3)	
C2	0.70993 (16)	0.28903 (12)	0.10401 (10)	0.0551 (3)	
C3	0.8843 (2)	0.29836 (17)	0.08793 (13)	0.0797 (5)	
H3A	0.9424	0.3256	0.0208	0.096*	
C4	0.9738 (2)	0.26921 (19)	0.16647 (15)	0.0924 (6)	
H4A	1.0904	0.2768	0.1532	0.111*	
C5	0.8877 (2)	0.22822 (18)	0.26596 (15)	0.0870 (5)	
H5A	0.9469	0.2089	0.3204	0.104*	
C6	0.71649 (18)	0.21556 (15)	0.28577 (12)	0.0686 (4)	
H6A	0.6615	0.1860	0.3533	0.082*	
C7	0.6255 (2)	0.32311 (13)	0.01581 (11)	0.0642 (4)	
H7A	0.6933	0.3485	−0.0488	0.077*	
C8	0.4047 (2)	0.35949 (15)	−0.07799 (12)	0.0796 (5)	
H8A	0.5003	0.3780	−0.1345	0.096*	
H8B	0.3306	0.4368	−0.0778	0.096*	
C9	0.30892 (18)	0.26118 (13)	−0.09649 (10)	0.0607 (4)	
C10	0.2035 (2)	0.29393 (18)	−0.16839 (13)	0.0834 (5)	
H10A	0.1899	0.3779	−0.2039	0.100*	
C11	0.1182 (3)	0.2036 (2)	−0.18815 (17)	0.1083 (7)	
H11A	0.0475	0.2272	−0.2368	0.130*	
C12	0.1365 (3)	0.0791 (2)	−0.13670 (17)	0.1025 (6)	
H12A	0.0798	0.0180	−0.1507	0.123*	
C13	0.2379 (2)	0.04685 (17)	−0.06566 (15)	0.0885 (5)	
H13A	0.2496	−0.0370	−0.0297	0.106*	
C14	0.3242 (2)	0.13565 (14)	−0.04556 (11)	0.0695 (4)	
H14A	0.3944	0.1108	0.0033	0.083*	
C15	0.35020 (15)	0.19827 (12)	0.32539 (9)	0.0511 (3)	
C16	0.32423 (16)	0.07005 (13)	0.36722 (10)	0.0559 (3)	
C17	0.22163 (19)	0.03899 (15)	0.46347 (11)	0.0703 (4)	
H17A	0.2019	−0.0454	0.4928	0.084*	
C18	0.1483 (2)	0.13004 (18)	0.51657 (12)	0.0802 (5)	
H18A	0.0799	0.1068	0.5813	0.096*	
C19	0.1753 (2)	0.25461 (17)	0.47477 (12)	0.0782 (5)	
H19A	0.1247	0.3155	0.5114	0.094*	
C20	0.27753 (18)	0.29204 (14)	0.37815 (11)	0.0625 (4)	
C21	0.3092 (3)	0.43053 (15)	0.33367 (15)	0.0877 (5)	
H21A	0.3848	0.4336	0.2667	0.105*	
C22	0.1524 (4)	0.5088 (2)	0.3115 (3)	0.1691 (13)	
H22A	0.1784	0.5951	0.2822	0.254*	
H22B	0.0705	0.5047	0.3744	0.254*	
H22C	0.1064	0.4775	0.2636	0.254*	
C23	0.4053 (6)	0.4786 (3)	0.3972 (3)	0.1996 (18)	
H23A	0.4256	0.5657	0.3664	0.299*	
H23B	0.5122	0.4291	0.4003	0.299*	
H23C	0.3401	0.4719	0.4657	0.299*	
C24	0.40181 (19)	−0.03072 (14)	0.30860 (13)	0.0717 (4)	
H24A	0.5049	0.0004	0.2614	0.086*	
C25	0.4532 (3)	−0.15552 (19)	0.3797 (2)	0.1265 (8)	
H25A	0.5297	−0.1403	0.4201	0.190*	
H25B	0.5087	−0.2145	0.3388	0.190*	
H25C	0.3535	−0.1901	0.4247	0.190*	
C26	0.2816 (3)	−0.0540 (2)	0.24438 (15)	0.1028 (6)	
H26A	0.2491	0.0247	0.2003	0.154*	
H26B	0.1820	−0.0891	0.2891	0.154*	
H26C	0.3375	−0.1123	0.2030	0.154*	

### Atomic displacement parameters (Å^2^)

**Table:**

	*U*^11^	*U*^22^	*U*^33^	*U*^12^	*U*^13^	*U*^23^
N1	0.0752 (8)	0.0591 (7)	0.0527 (7)	−0.0122 (6)	−0.0189 (6)	−0.0042 (5)
N2	0.0467 (6)	0.0823 (8)	0.0450 (6)	−0.0171 (5)	−0.0106 (5)	−0.0034 (5)
C1	0.0459 (7)	0.0537 (7)	0.0546 (8)	−0.0083 (5)	−0.0080 (6)	−0.0113 (6)
C2	0.0547 (8)	0.0551 (7)	0.0545 (8)	−0.0105 (6)	−0.0037 (6)	−0.0124 (6)
C3	0.0557 (9)	0.1029 (12)	0.0750 (10)	−0.0197 (8)	0.0063 (8)	−0.0185 (9)
C4	0.0484 (9)	0.1297 (15)	0.0985 (14)	−0.0193 (9)	−0.0079 (9)	−0.0231 (12)
C5	0.0588 (10)	0.1167 (14)	0.0885 (12)	−0.0088 (9)	−0.0277 (9)	−0.0160 (10)
C6	0.0541 (9)	0.0904 (10)	0.0607 (9)	−0.0129 (7)	−0.0157 (7)	−0.0078 (7)
C7	0.0755 (10)	0.0643 (8)	0.0498 (8)	−0.0217 (7)	−0.0010 (7)	−0.0079 (6)
C8	0.1094 (13)	0.0708 (9)	0.0597 (9)	−0.0189 (9)	−0.0332 (9)	0.0041 (7)
C9	0.0649 (9)	0.0695 (9)	0.0463 (7)	0.0001 (6)	−0.0108 (6)	−0.0130 (6)
C10	0.0935 (12)	0.0892 (11)	0.0719 (10)	0.0053 (9)	−0.0353 (9)	−0.0155 (9)
C11	0.1071 (16)	0.1313 (19)	0.1084 (16)	0.0040 (13)	−0.0600 (13)	−0.0408 (14)
C12	0.0989 (15)	0.1114 (16)	0.1186 (17)	−0.0122 (12)	−0.0366 (13)	−0.0503 (13)
C13	0.0992 (13)	0.0763 (11)	0.0974 (13)	−0.0104 (9)	−0.0261 (11)	−0.0250 (10)
C14	0.0754 (10)	0.0696 (9)	0.0664 (9)	−0.0040 (7)	−0.0203 (8)	−0.0155 (7)
C15	0.0411 (6)	0.0669 (8)	0.0459 (7)	−0.0101 (5)	−0.0108 (5)	−0.0083 (6)
C16	0.0439 (7)	0.0666 (8)	0.0578 (8)	−0.0123 (6)	−0.0102 (6)	−0.0102 (6)
C17	0.0667 (9)	0.0768 (10)	0.0618 (9)	−0.0243 (7)	−0.0055 (7)	−0.0009 (7)
C18	0.0742 (10)	0.1087 (14)	0.0527 (9)	−0.0260 (9)	0.0057 (7)	−0.0138 (9)
C19	0.0754 (10)	0.0944 (12)	0.0675 (10)	−0.0056 (8)	−0.0022 (8)	−0.0332 (9)
C20	0.0596 (8)	0.0686 (8)	0.0609 (8)	−0.0075 (6)	−0.0127 (7)	−0.0148 (7)
C21	0.1018 (13)	0.0631 (9)	0.0948 (12)	−0.0060 (9)	−0.0115 (10)	−0.0175 (9)
C22	0.152 (2)	0.0931 (16)	0.229 (3)	0.0249 (16)	−0.043 (2)	0.0145 (19)
C23	0.305 (5)	0.0993 (17)	0.238 (4)	−0.079 (2)	−0.137 (4)	−0.013 (2)
C24	0.0598 (9)	0.0696 (9)	0.0848 (11)	−0.0114 (7)	−0.0024 (8)	−0.0220 (8)
C25	0.147 (2)	0.0824 (13)	0.158 (2)	0.0248 (12)	−0.0558 (17)	−0.0354 (14)
C26	0.0970 (13)	0.1335 (17)	0.0957 (13)	−0.0102 (11)	−0.0151 (11)	−0.0607 (12)

### Geometric parameters (Å, °)

**Table:**

N1—C7	1.2659 (18)	C14—H14A	0.9300
N1—C8	1.4557 (17)	C15—C20	1.3921 (19)
N2—C1	1.3646 (16)	C15—C16	1.4006 (18)
N2—C15	1.4307 (16)	C16—C17	1.3831 (19)
N2—H2A	0.8600	C16—C24	1.511 (2)
C1—C6	1.3973 (18)	C17—C18	1.372 (2)
C1—C2	1.4134 (18)	C17—H17A	0.9300
C2—C3	1.389 (2)	C18—C19	1.366 (2)
C2—C7	1.449 (2)	C18—H18A	0.9300
C3—C4	1.364 (2)	C19—C20	1.393 (2)
C3—H3A	0.9300	C19—H19A	0.9300
C4—C5	1.379 (2)	C20—C21	1.517 (2)
C4—H4A	0.9300	C21—C22	1.475 (3)
C5—C6	1.367 (2)	C21—C23	1.492 (3)
C5—H5A	0.9300	C21—H21A	0.9800
C6—H6A	0.9300	C22—H22A	0.9600
C7—H7A	0.9300	C22—H22B	0.9600
C8—C9	1.495 (2)	C22—H22C	0.9600
C8—H8A	0.9700	C23—H23A	0.9600
C8—H8B	0.9700	C23—H23B	0.9600
C9—C14	1.379 (2)	C23—H23C	0.9600
C9—C10	1.380 (2)	C24—C26	1.513 (2)
C10—C11	1.377 (3)	C24—C25	1.530 (3)
C10—H10A	0.9300	C24—H24A	0.9800
C11—C12	1.374 (3)	C25—H25A	0.9600
C11—H11A	0.9300	C25—H25B	0.9600
C12—C13	1.348 (3)	C25—H25C	0.9600
C12—H12A	0.9300	C26—H26A	0.9600
C13—C14	1.371 (2)	C26—H26B	0.9600
C13—H13A	0.9300	C26—H26C	0.9600
			
C7—N1—C8	118.36 (13)	C17—C16—C15	117.56 (13)
C1—N2—C15	124.45 (11)	C17—C16—C24	120.99 (13)
C1—N2—H2A	117.8	C15—C16—C24	121.43 (12)
C15—N2—H2A	117.8	C18—C17—C16	121.44 (14)
N2—C1—C6	121.68 (12)	C18—C17—H17A	119.3
N2—C1—C2	119.94 (12)	C16—C17—H17A	119.3
C6—C1—C2	118.37 (12)	C19—C18—C17	120.24 (14)
C3—C2—C1	118.26 (13)	C19—C18—H18A	119.9
C3—C2—C7	118.80 (13)	C17—C18—H18A	119.9
C1—C2—C7	122.93 (12)	C18—C19—C20	121.11 (15)
C4—C3—C2	122.75 (15)	C18—C19—H19A	119.4
C4—C3—H3A	118.6	C20—C19—H19A	119.4
C2—C3—H3A	118.6	C15—C20—C19	117.74 (14)
C3—C4—C5	118.60 (15)	C15—C20—C21	121.76 (13)
C3—C4—H4A	120.7	C19—C20—C21	120.49 (14)
C5—C4—H4A	120.7	C22—C21—C23	114.9 (2)
C6—C5—C4	120.95 (16)	C22—C21—C20	112.11 (18)
C6—C5—H5A	119.5	C23—C21—C20	111.35 (17)
C4—C5—H5A	119.5	C22—C21—H21A	105.9
C5—C6—C1	121.06 (14)	C23—C21—H21A	105.9
C5—C6—H6A	119.5	C20—C21—H21A	105.9
C1—C6—H6A	119.5	C21—C22—H22A	109.5
N1—C7—C2	125.85 (13)	C21—C22—H22B	109.5
N1—C7—H7A	117.1	H22A—C22—H22B	109.5
C2—C7—H7A	117.1	C21—C22—H22C	109.5
N1—C8—C9	113.42 (12)	H22A—C22—H22C	109.5
N1—C8—H8A	108.9	H22B—C22—H22C	109.5
C9—C8—H8A	108.9	C21—C23—H23A	109.5
N1—C8—H8B	108.9	C21—C23—H23B	109.5
C9—C8—H8B	108.9	H23A—C23—H23B	109.5
H8A—C8—H8B	107.7	C21—C23—H23C	109.5
C14—C9—C10	117.49 (14)	H23A—C23—H23C	109.5
C14—C9—C8	122.37 (13)	H23B—C23—H23C	109.5
C10—C9—C8	120.13 (13)	C16—C24—C26	110.94 (13)
C11—C10—C9	120.70 (17)	C16—C24—C25	112.51 (15)
C11—C10—H10A	119.7	C26—C24—C25	109.81 (16)
C9—C10—H10A	119.7	C16—C24—H24A	107.8
C12—C11—C10	120.62 (18)	C26—C24—H24A	107.8
C12—C11—H11A	119.7	C25—C24—H24A	107.8
C10—C11—H11A	119.7	C24—C25—H25A	109.5
C13—C12—C11	118.89 (18)	C24—C25—H25B	109.5
C13—C12—H12A	120.6	H25A—C25—H25B	109.5
C11—C12—H12A	120.6	C24—C25—H25C	109.5
C12—C13—C14	121.10 (18)	H25A—C25—H25C	109.5
C12—C13—H13A	119.5	H25B—C25—H25C	109.5
C14—C13—H13A	119.5	C24—C26—H26A	109.5
C13—C14—C9	121.20 (15)	C24—C26—H26B	109.5
C13—C14—H14A	119.4	H26A—C26—H26B	109.5
C9—C14—H14A	119.4	C24—C26—H26C	109.5
C20—C15—C16	121.90 (12)	H26A—C26—H26C	109.5
C20—C15—N2	119.19 (12)	H26B—C26—H26C	109.5
C16—C15—N2	118.90 (12)		
			
C15—N2—C1—C6	4.9 (2)	C8—C9—C14—C13	−178.51 (16)
C15—N2—C1—C2	−176.32 (12)	C1—N2—C15—C20	88.21 (16)
N2—C1—C2—C3	−178.86 (12)	C1—N2—C15—C16	−92.83 (15)
C6—C1—C2—C3	−0.01 (19)	C20—C15—C16—C17	0.5 (2)
N2—C1—C2—C7	1.0 (2)	N2—C15—C16—C17	−178.38 (11)
C6—C1—C2—C7	179.87 (12)	C20—C15—C16—C24	179.15 (13)
C1—C2—C3—C4	−0.6 (2)	N2—C15—C16—C24	0.22 (18)
C7—C2—C3—C4	179.48 (16)	C15—C16—C17—C18	−0.3 (2)
C2—C3—C4—C5	0.3 (3)	C24—C16—C17—C18	−178.90 (14)
C3—C4—C5—C6	0.7 (3)	C16—C17—C18—C19	0.1 (3)
C4—C5—C6—C1	−1.4 (3)	C17—C18—C19—C20	−0.2 (3)
N2—C1—C6—C5	179.82 (14)	C16—C15—C20—C19	−0.6 (2)
C2—C1—C6—C5	1.0 (2)	N2—C15—C20—C19	178.32 (12)
C8—N1—C7—C2	179.73 (13)	C16—C15—C20—C21	178.58 (13)
C3—C2—C7—N1	−178.73 (13)	N2—C15—C20—C21	−2.5 (2)
C1—C2—C7—N1	1.4 (2)	C18—C19—C20—C15	0.4 (2)
C7—N1—C8—C9	124.20 (15)	C18—C19—C20—C21	−178.78 (15)
N1—C8—C9—C14	−19.0 (2)	C15—C20—C21—C22	115.1 (2)
N1—C8—C9—C10	162.48 (14)	C19—C20—C21—C22	−65.7 (3)
C14—C9—C10—C11	−0.3 (2)	C15—C20—C21—C23	−114.7 (3)
C8—C9—C10—C11	178.24 (18)	C19—C20—C21—C23	64.5 (3)
C9—C10—C11—C12	−0.1 (3)	C17—C16—C24—C26	87.45 (18)
C10—C11—C12—C13	0.7 (3)	C15—C16—C24—C26	−91.11 (17)
C11—C12—C13—C14	−1.0 (3)	C17—C16—C24—C25	−36.0 (2)
C12—C13—C14—C9	0.7 (3)	C15—C16—C24—C25	145.44 (16)
C10—C9—C14—C13	0.0 (2)		

### Hydrogen-bond geometry (Å, °)

**Table:**

*D*—H···*A*	*D*—H	H···*A*	*D*···*A*	*D*—H···*A*
N2—H2A···N1	0.86	2.03	2.7113 (15)	136
